# Correspondence

**Published:** 1916-07

**Authors:** 


### CORRESPONDENCE.

This department is intended for the presentation of news and views not coming within the scope of other departments, and particularly to afford opportunity for discussion and criticism of views elsewhere expressed.

---

### American Association for Labor Legislation.

131 East 23d St., New York City

May 29, 1916.

Dr. A. L. Benedict, Editor,
Buffalo Medical Journal,
Dear Sir:—

Your very excellent editorial on the problem of the Independent poor which appeared in your Journal for May, leads me to think that you will be interested in health insurance as a method of providing medical care for this group.

In such an insurance system as we are proposing it will be possible to provide the necessary medical care, including hospital and nursing attendance when needed, in return for a small weekly contribution divided among the employer, the employee and the State.

In addition, other benefits such as a weekly payment equal to two-thirds of wages for not more than 26 weeks in a year would be available. Of course you realize that the absence of income during illness of the wage earner is often a calamity to the family and retards his own recovery.

The sections (8-14) which provide for the administration of medical benefit will prove of special interest to medical men.

Trusting that you will use your influence to bring this matter to the attention of the medical profession, I am,

Very truly yours,

JOHN B. ANDREWS,

Secretary.

We are heartily in sympathy with the general principles underlying the effort to obtain insurance of this kind but there are certain details which ought to be carefully considered in advance. For various reasons, we are inclined to deprecate special legislation in favor of manual laborers, particularly when the amount of wages is not considered. Without regard to the matter at issue, we consider it undesirable in this country to do anything that tends to create a fixed laboring class, that is to say, that tends to limit the meaning of the words work and labor and to establish degrees of dignity in performing useful work or that tends to stratify society. With particular reference to the present movement, we feel that, on the one hand, manual labor in the broad sense is just as intelligent and thrifty as clerical; on the other hand that clerical or professional or any other kind of work, well performed and meeting a genuine need of the community, is equally deserving of issistance if assistance is required at all. Moreover, the distinction between manual and non-manual labor is very difficult to define, except according to arbitrary standards. For example, there are surgeons, dentists and medical specialists who not only use more actual manual skill but more muscle than many who would be classified as manual laborers but whose hand work is mainly confined to the manipulation of levers, and whose skill is essentially mental.

We appreciate the great practical difficulties of applying to home and "casual" employes, the principle of responsibility by the employer, as in the case of large corporations or firms employing large numbers of employes quite steadily. An attempt in this direction would undoubtedly be to the disadvantage of the employe, since it would impose almost prohibitive responsibility on the prospective employer. Yet it is the domestic servant and the transient worker of either sex that needs the protection of insurance, even more than the trained mechanic, more regularly employed.

We cannot without further enlightenment, appreciate how the kind of work that a person does, has any bearing on his need of insurance against disaster or, to put the matter in the opposite way, how the community as a whole, needs to be protected against the loss of a worker and against the pauperization of an individual or family, for certain kinds of workers and not for others. We appreciate very clearly the principles that the community does owe to the fullest degree practicable, protection against disease and accident to all its members; and that it owes to those who would be financially crippled by disease and accident, not only care and support, but care and support on a self-respecting basis and in such a way that the integrity of the home and family shall not be sacrificed. In other words, the community should provide, not merely the hospital, the poor house and the asylum, but some sort of insurance that should enable the family to continue as a unit and on approximately the same scale of living which its chief wage earner has already established, as what the world owes him. We can not conceive of this debt of the community to the individual, as being increased because the particular individual does any particular kind of work and is usually paid in any particular way, nor diminished because he does some other kind of work or gets his pay according to some other method. Obviously a point is reached at which the individual does not absolutely require insurance or may be safely allowed to suffer loss if he has not had the foresight to secure it of his own initiative, and at which insurance at the average rate of income is not advisable as a compulsory matter, especially if the income does not depend directly on the health and strength of the nominal wage earner. Off hand, an income of $1200 a year—or more for laborers technically classed as manual—seems rather a high limit for compulsory insurance on the combined basis of deduction from earnings, and assessment on the employer and the state. On the other hand, if the state can insure generally, at a lower rate and more safely than a private company, there is an obvious advantage in making this provision one of the functions of government and, if undertaken by the state at all, it should be compulsory—or rather automatic—and should apply to all who could conceivably be reduced to pauperism by a failure of earning capacity.

